# Home-based self-management using Traditional Chinese Medicine techniques: empowerment and risks in breast cancer survivors’ fulfillment of health-deviation self-care requisites

**DOI:** 10.1007/s11764-025-01808-2

**Published:** 2025-05-09

**Authors:** Fei-Yi Zhao, Peijie Xu, Gerard A. Kennedy, Li-Ping Yue, Wen-Jing Zhang, Yan-Mei Wang, Yuen-Shan Ho, Qiang-Qiang Fu, Russell Conduit

**Affiliations:** 1 Department of Nursing, School of International Medical Technology, Shanghai Sanda University, Shanghai, 201209 People’s Republic of China; 2 Sydney School of Health Sciences, Faculty of Medicine and Health, The University of Sydney, Camperdown, NSW 2050 Australia; 3 School of Health and Biomedical Sciences, RMIT University, Bundoora, VIC 3083 Australia; 4 Shanghai Municipal Hospital of Traditional Chinese Medicine, Shanghai University of Traditional Chinese Medicine, Shanghai, 200071 People’s Republic of China; 5 School of Computing Technologies, RMIT University, Melbourne, VIC 3000 Australia; 6 School of Nursing, Faculty of Health and Social Sciences, The Hong Kong Polytechnic University, Hong Kong, SAR People’s Republic of China; 7 Yangpu Hospital, School of Medicine, Tongji University, Shanghai, 200090 People’s Republic of China

**Keywords:** Complementary and alternative medicine, Cancer rehabilitation, Integrative care, Self-treatment, Self-administration, Patient experiences, Qualitative study, Ethnography

## Abstract

**Background and aim:**

Traditional Chinese Medicine (TCM)–based self-care is widely practiced among cancer survivors worldwide, particularly in China. For Chinese breast cancer survivors (BCSs), it embodies both an expression of ethnomedical cultural identity and a means of health self-empowerment. However, this practice often occurs without professional supervision, posing potential risks. This study aims to explore BCSs’ experiences and perspectives to elucidate the purposes and influencing factors behind their home-based TCM self-care engagement, thereby informing future optimization strategies.

**Methods:**

A focused ethnographic design was utilized, with a research framework integrating the COM-B model and Theoretical Domains Framework (TDF). Results were mapped onto constructs of a behavioral wheel derived from the COM-B/TDF matrix. Data analysis followed conventional qualitative content analysis procedures.

**Results:**

Participants viewed TCM-based self-care as a complement to rather than a substitute for standard care, primarily for relapse prevention. Facilitators of this practice included (1) strong cultural beliefs and confidence in ethnic medicine, (2) a sense of health responsibility, (3) heightened internal health locus of control, (4) prior beneficial experiences, (5) incentives from online key opinion leaders and fellow survivors, (6) support from family and peers, and (7) reduced time and financial costs. Barriers were (1) insufficient TCM knowledge and skills and (2) uncertainty about efficacy; and (3) safety concerns. Additionally, (1) the home-based treatment setting and (2) the immature internet-based TCM nurse service acted as both barriers and facilitators.

**Conclusions:**

The interrelated facilitators and barriers underscore that BCSs’ home-based TCM self-care constitutes a complex medical-sociological issue involving cultural, economic, information communication, and healthcare service delivery dimensions. Only a minority of survivors are aware of the possible adverse medical consequences. Within a patient-centered framework, healthcare providers must identify the unique health-deviation self-care requisites of BCSs in a culturally sensitive manner, and partner with them in their self-health management, such as telemonitoring their home-based TCM self-care using eHealth technologies. There is also an urgent need to develop clinical guidelines or expert consensus to support these practices.

**Implications for Cancer Survivors:**

BCSs' TCM self-care represents an intentional health autonomy strategy beyond conventional biomedical dominance, necessitating professional supervision to equilibrate health self-empowerment and iatrogenic risk.

**Supplementary Information:**

The online version contains supplementary material available at https://doi.org/10.1007/s11764-025-01808-2.

## Background

### Widespread TCM-based self-care among breast cancer survivors

Breast cancer was the second leading cause of cancer incidence globally in 2022, constituting 11.6% of all cases and remaining the most frequently diagnosed cancer in women [1]. An estimated 2.3 million new cases occur annually [1]. Self-management is an integral aspect of cancer survivorship care [2], serving to address treatment-related concerns, enhance well-being [3], and boost self-efficacy [2].

Data from 14 European countries reveal that complementary and alternative medicine (CAM) usage among cancer survivors varies from 14.8 to 73.1% [4]. Breast cancer survivors (BCSs) exhibit a relatively higher CAM utilization rate (45%) compared to other cancer survivors [4]. CAM services are accessed through both professional healthcare providers and self-help approaches [5], with evidence suggesting a preference for self-directed practices among BCSs [4]. The WHO and the UK National Health Service define “self-care” as learned behaviors undertaken by individuals in response to perceived health changes to improve well-being, prevent disease or deterioration, and manage chronic conditions [6]. Thus, self-directed CAM practice by BCSs can be considered a form of self-care.

Among various CAM modalities, Traditional Chinese Medicine (TCM) is highly favored by BCSs. In the United States, acupuncture ranks among the top three most commonly used CAM therapies by BCSs [7]. In China, 98% of BCSs reported using at least one CAM therapy, with 86.4% utilizing Chinese herbal medicine [8]. This is unsurprising, given that TCM is the most popular CAM in China [9], with approximately 60% of residents having used TCM services at least once [10] and TCM healthcare agencies accounting for 20% of national healthcare provision [11].

Clinical trials have demonstrated the feasibility and efficacy of professionally directed yet self-administered TCM practices—such as self-acupressure, self-acupuncture, and home-based Qigong—in managing cancer-related fatigue [12–14], vasomotor symptoms [15], sleep disturbances [13, 16], and constipation [16]. However, many Chinese BCSs rely primarily on their own understanding of TCM for self-care at home [6]. Notably, similar TCM-based self-care practice has been observed among cancer survivors in regions outside Asia, including the United States, Europe, and Australia [17]. (***Notes***: The term “TCM-based self-care practice,” as used herein, specifically refers to “the scenario where a BCS uses TCM independently, without professional guidance or oversight, to manage illness or promote health.”).

This prompts critical questions: Are BCSs aware of the risks of unsupervised TCM self-care? What motivates them to engage in these practices? What are their experiences? Research on these issues remains scarce. Nevertheless, educational or other interventional strategies are needed to minimize these potentially risky behaviors. Developing such strategies requires an in-depth understanding of (1) BCSs’ reasons for engaging in home-based TCM self-care, and (2) the facilitators and barriers influencing these practices. These constitute the key objectives and rationale of this study.

### Theoretical framework, model, and research paradigm

The Capability, Opportunity, and Motivation Behavior (COM-B) model, proposed by Michie et al., offers a comprehensive framework for understanding behavior [18]. This pragmatic model conceptualizes behavior as part of an interactive system [19], postulating that for a behavior (B) to occur, an individual needs to be physically and psychologically capable (C) of utilizing social and physical opportunities (O) through motivators (M) that are either automatic or reflective [18]. Motivation serves as the central component, influenced by capability and opportunity [20], with all elements interacting to produce behavior [19]. Unlike single-theory approaches, the COM-B model’s integration of multiple distinct explanatory components [21] systematically identifies behavioral barriers and facilitators [22], making it particularly applicable to this study.

In our study, the COM-B model was also integrated with the Theoretical Domains Framework (TDF) to structure the interview guide and data analysis. This established health research approach [20, 23, 24] effectively captures behavioral drivers through framework analysis [23]. The TDF integrates psychological theories (e.g., *Theory of Planned Behavior*, *Social Cognitive Theory*) and theoretical constructs (e.g., *Goal Priority*, *Competence*) [25] across fourteen domains aligned with COM-B components [20], enabling granular behavioral analysis [23] of proximal determinants [24].

We adopted focused ethnography as the research paradigm. TCM, as a cultural system rooted in traditional Chinese and Sinicized foreign influences [26], embodies distinct indigenous characteristics with philosophical, cognitive, and developmental trajectory reflecting Chinese cultural traditions [27]. This cultural embeddedness profoundly impacts local patients’ medical behaviors and clinical decision-making [28], paralleling how cultural folk remedies shape self-care among cancer survivors in other regions (e.g., U.S. Midwest, Philippines) [6]. These cross-cultural variations underscore self-care’s dual medical-social nature, requiring contextual interpretation. Ethnography’s emphasis on social contextual analysis [29] makes it ideal for examining patients’ thoughts, behaviors, and feelings throughout their healthcare journey and how healthcare interventions are influenced by patients’ culture context [30]. A focused ethnographic design was selected over other ethnographic methods for two methodological merits: (1) focused analysis of shared experiences in predetermined contexts [31]; (2) less fieldwork and intermittent observation compared to traditional ethnography [31], minimizing participant burden and accommodating cancer survivors’ physical limitations—a key ethical consideration.

## Materials and methods

### Study design and participants

Focused ethnography, characterized by a brief and intensive data collection period, employs methods such as observational fieldwork, interviews, and/or document reviews [32]. Thus, participants’ homes were selected as study sites. We recruited BCSs through WeChat groups affiliated with two cancer rehabilitation NGOs in Shanghai, China. Inclusion criteria required participants to (1) be female, first-diagnosis BCSs without current recurrence/metastasis; (2) have completed anticancer treatment for at least 6 months; (3) have practiced self-directed TCM self-care for a minimum of 2 weeks (regardless of current use); and (4) possess cognitive capacity for independent interviews. BCSs were excluded if (1) their TCM self-care was professionally supervised, (2) they had comorbid malignancies or severe physical/mental conditions, or (3) their TCM use was unrelated to breast cancer management. TCM practices such as fire cupping or Gua-sha performed with the assistance of a family member (non-medical personnel) were also considered self-care.

Following comprehensive study briefings, participants provided written informed consent prior to the two-phase investigation: fieldwork observation and semi-structured interviews. The study complied with the *Consolidated Criteria for Reporting Qualitative Research* (COREQ) [33] and *Standards for Reporting Qualitative Research* (SRQR) [34] to ensure rigorous structuring and reporting.

### Procedures

The study was conducted between July and October 2024.

#### Fieldwork phase

Research assistants recorded participants’ TCM self-care practices at home using portable cameras. Following ethnographic principles [30], they minimized interaction while recording observations, supplementing video data with fieldnotes capturing sensory details and unconscious behaviors. We recruited BCSs using purposive [35] and snowball [36] sampling methods and selected candidate households using a maximum variation strategy [37] to ensure diverse TCM techniques were observed. Participants were informed that the study focused on identifying facilitators and barriers of their TCM-based self-care, rather than the behavior’s validity, to mitigate the “Hawthorne effect.”

#### Interview phase

The interview guide, initially crafted based on the COM-B model and TDF following a literature review, was refined after pilot interviews with three potential candidates (Appendix 1). Research assistants followed participants’ thought processes, allowing deviations for follow-up questions to gain deeper insights. The semi-structured approach maximized opportunities for participant self-expression [38]. All observed participants during fieldwork were interviewed, and snowball sampling continued to enrich the study data. Data saturation was determined using Francis et al.’s criteria [39], with an initial sample of at least ten interviews and a stopping criterion (no new themes in three consecutive interviews). Interviews, conducted by pairs of research assistants, focused on understanding participants’ TCM self-care objectives and experiences, and explored reasons for cessation if applicable. Family members were permitted to be present to provide supplementary information. Fieldnotes continued to be used to supplement interview data, and detailed demographic and clinical data were collected at the beginning of each interview.

#### Qualifications of research assistants

The four research assistants were *Master of Nursing* (*MNurs*) students, with two holding TCM-based *Bachelor of Nursing Science* (*BNS*) degrees and having personal TCM experience. Fieldwork was conducted independently by each assistant to maintain continuous, uninterrupted footage. Interviews employed a 2:1 format, with one assistant posing questions and another recording the conversation and noting non-verbal cues. Each pair included one TCM-knowledgeable assistant and one without, facilitating accurate TCM understanding and preventing effectiveness exaggeration, thus addressing interviewer reliability and bias issues [40].

#### Session duration

To minimize disruption and consider the time required for each TCM technique, fieldwork was capped at 30 min per session, and interviews at 40 min. If a participant demonstrated her TCM-based self-care during the interview, research assistants recorded the process with consent. Following each session, assistants completed reflective journals to inform and refine subsequent fieldwork and interviews.

### Data collection and analysis

Data collection and analysis proceeded concurrently. Interview audio recordings, textualized into Chinese using a speech-to-text application (Xunfei Hearing, version 7.0.3904), were cleaned and validated by four interviewers. Transcriptions were translated into English by a NAATI-Certified translator (P-X). Video materials demonstrating participant’s TCM self-care were incorporated into the fieldwork data for analysis. Qualitative content analysis (QCA) [41] was used to establish coding units within a coding frame derived from the transcripts, processed separately by two analysts (LP-Y and FY-Z). Given the study utilized open-ended probes rather than extending pre-existing theories on BCSs’ self-care or conducting manifest content analysis of keywords [42], conventional QCA was employed. Two analysts independently sought statements describing experiences related to the research objectives and extracted meaning units. QCA accommodates both manifest and latent meaning, allowing for deductive, inductive, or combined approaches [43]. The integration of manifest and latent content analysis, due to their varying depth and abstraction, yields more insightful findings, with a focus to compare the meaning units with regard to similarities and dissimilarities [44]. To enhance trustworthiness, researcher triangulation [44] was implemented. Five researchers with interdisciplinary backgrounds (QQ-F, YM-W, P-X, LP-Y, and YS-H) grouped similar meaning units into new, recoded groups (i.e., condensed meaning units) close to the text and related to the study objective. These condensed meaning units were then iteratively scrutinized, reflected upon and discussed by all researchers in order to uncover new, more abstract dimensions and be further condensed into different sub-themes and theme denoting the hidden meaning of the text through a deductive process. Each stage’s results were cross-verified against the research questions to ensure accuracy and reliability. Discrepancies were resolved through team discussions.

The fieldwork videos were subjected to frame-by-frame analysis using video editing software (JianYing, version 6.3.0.12011). Two licensed TCM practitioners with over 10 years of clinical experience (FY-Z and WJ-Z) reviewed the videos to assess participants’ execution of TCM techniques, identifying any apparent ineffective or erroneous practices and potential safety risks. Insights gained from the video analysis, alongside fieldnotes and reflective journals, were systematically integrated into the coding process.

### Methodological rigor

Based on Lincoln and Guba’s “Four-Dimensions Criteria” (i.e., Credibility, Dependability, Confirmability, and Transferability) [45], we implemented a range of measures to establish trustworthiness and minimize bias (Appendix 2). Additionally, self-reflection and a reflexive approach [46] were rigorously applied throughout the study to critically prompt how our cultural, professional, and personal backgrounds influenced the research process [29].

### Ethics approval and consent to participate

All original audio and video recordings were deleted after transcription. Participants were assigned unique identifiers (P1, P2, P3, etc.) to maintain anonymity in transcripts and this paper. Identifiable information, including addresses, was removed. Written informed consent for participation and publication of anonymized data was obtained from all participants. The study protocol received ethical approval from the Human Research Ethics Committee (HREC) of Shanghai Sanda University (No. 2024007).

## Results

Due to privacy concerns and other considerations, only seven BCSs consented to home-based field observations, while over 40 candidates expressed interest in interviews. Thematic redundancy was reached after the 23rd interview, with no new information emerging in the subsequent three, rendering the final total at 26 interviews. Participants averaged 53.1 ± 7.3 years (Appendix 3). All had surgery, with most also receiving radiotherapy and/or chemotherapy. Currently, 88.5% of participants practiced TCM-based self-care, though three discontinued due to concerns about efficacy and/or safety. The primary reasons for using TCM-based self-care included relapse prevention (100%) and immune system enhancement (84.6%). Additionally, 73.1% sought to alleviate radiotherapy/chemotherapy-related side effects or manage other symptoms during postoperative/recovery period, particularly fatigue (69.2%), sleep disturbances (61.5%), and negative emotions (50.0%). The core findings of the study, derived from the QCA of the raw data obtained from interviews and fieldworks (Appendix 4), are presented as follows.

### Motivation domain

#### Deep-seated cultural belief and confidence in ethnic medicine

Participants exhibited substantial confidence in TCM, regarding it as a fundamental component of Chinese traditional culture and a cultural heritage inherited from their ancestors. They acknowledged that their TCM use was heavily shaped by this deep-rooted cultural belief in ethnic medicine.

TCM has survived for thousands of years, so it must have something special about it. As Chinese individuals, it is natural for us to trust our own traditional medicine. (P16).

I have always been a big fan of TCM. It’s the medicine of the Chinese nation. Before Western medicine existed, our ancestors relied on TCM to heal and save lives. Even before I was diagnosed with breast cancer, I was drinking TCM herbal teas for health. After getting sick, I immediately sought TCM for recovery and well-being. (P25).

#### Previous beneficial experiences

Some BCSs had encountered the efficacy of TCM either prior to their cancer diagnosis or during the perioperative period. These favorable clinical experiences significantly influenced their subsequent adoption of TCM-based self-care practices during the cancer recovery journey.

I developed lymphedema after mastectomy. The nurses not only gave me elastic bandages, did lymphatic drainage, but they also applied Danggui Bohe balm (a Chinese herbal ointment) near the wound area, and did moxibustion on me. The swelling then improved a lot. So now, I often do moxibustion at home myself. I think it helps with my insomnia and fatigue. (P21).

I used to have severe allergic rhinitis, and even steroids did not help. I got worried about the side effects of the steroids and stopped using them. I then tried acupuncture instead, and it really improved my symptoms. Since then, I have been a big believer in acupuncture. For the nausea and vomiting from chemotherapy, I also went for acupuncture. I even bought the smallest thumb-tack needles online to use for self-treatment. (P9).

#### Drive of health responsibility and duties

One participant articulated that engaging in TCM-based self-care practices fulfilled one’s “Health Responsibility.” She also discussed the interplay between individual rights and corresponding obligations within the context of self-care practices.

Cancer is a chronic illness. Individuals with chronic diseases need to take responsibility for their own health. Rights and duties are inextricably linked. Taking responsibility for your health is a duty, and once you fulfill that duty, you will feel a greater sense of control over your health, which is your right. Patients should have greater responsibility for their health than doctors do. (P12).

#### Desire for empowered self-health control (awakened/intensified internal health locus of control)

Despite many participants noting that their TCM-based self-care behaviors aimed to “fill the care gaps that Western medicine could not fully meet,” only one participant encapsulated this notion as a “sense of control over one’s own health.” She described her mindset prior to engaging in self-care as one of helplessness, which TCM-based self-care alleviated, thereby empowering her with more control over her health and destiny. This perspective can also be interpreted as an awakened or heightened internal health locus of control (HLOC).

CT scans, biopsies, surgeries, and chemotherapy…all of this left me feeling completely drained. My fate was always in someone else’s hands. It made me feel helpless. I wanted to have a sense of control over my own health. Using medical diet therapy of TCM and other self-care methods makes me feel like my health and destiny are in my own hands. (P18).

### Opportunity domain

#### Social support and peer experience sharing

Due to the limited discussions with oncologists and registered nurses regarding the use of TCM during consultations, BCSs had to seek TCM knowledge and skills related to managing post-cancer sequelae and facilitating recovery from other survivors, including relatives and friends. Consequently, their self-care behaviors were largely inspired by the personal experiences shared within their peer network and received substantial support from their families.

In our patient support group on WeChat, if someone has good tips on recovery, wellness, or medical care, they are always happy to share them. (P11).

My aunt is also a breast cancer patient. After chemotherapy, she lost her appetite. The nurse advised her to massage her abdomen and stimulate the acupoints there, which helped improve her appetite a bit. When she saw me struggling with my appetite, she shared this experience with me. (P24).

I heard other patients say they usually use techniques like Gua-sha, fire cupping, moxibustion, and Tai Chi at home, and that it works pretty well for them. Thus, I thought I should give it a try too. My family also supports this decision. (P8).

#### Social media influencers’ anticancer information dissemination

In addition to peer recommendations, endorsements by social media influencers also incentivize BCSs to engage in TCM-based self-care activities. These influencers can be broadly categorized into two types: (1) key opinion leaders (KOLs) who identify themselves as “TCM anticancer experts”; and (2) internet celebrities with substantial followers, often in the thousands, who are dedicated to sharing their anticancer experiences. (***Note***: Due to lack of authorization, the names of the influencers mentioned below are pseudonymized as *X*.)

One of the TikTok anchorwomen I follow, ‘Mrs. *X*’, is also a BCS who has not had a recurrence in five years. Her life is now just like that of a healthy person. I am a big fan of hers and hope to recover as well as she did. Therefore, I watch her Vlogs every day. I downloaded some of her Vlogs regarding TCM-based self-care and tried those methods at home myself. (P19).

Doctor *X *shares Vlogs on TikTok of him treating cancer patients, where he suggests some of them take Ganoderma lucidum nutraceuticals. The fact that patients travel from far away to see him shows just how skilled he is. In his pinned Vlog, Doctor *X* talks about how his TCM expertise is passed down through his family. So, I trust him a lot and follow his recommendations to purchase Ganoderma lucidum capsules online. (P7).

*Fieldnotes*: When we inquired if P7 verified the credentials of influencers she followed (such as Doctor *X*), how she determined whether these vlogs were merely a form of online marketing for nutraceuticals, and how she ascertained the suitability of the self-care techniques and products mentioned in the vlogs for her own use, P7 immediately retorted, “The people in the vlogs are also cancer survivors; they had good results using these techniques and products, so it doesn’t hurt to give it a try.” P7 deliberately avoided addressing our concerns regarding the influencer’s qualifications and the potential marketing tactics involved.

#### Reduced time and financial costs

The substantial costs associated with conventional cancer treatments impose a significant economic burden on BCSs. As a result, BCSs, especially those with financial constraints, often regard home-based self-care as a more affordable alternative to TCM care provided by hospitals.

Many imported molecularly targeted drugs and chemotherapy drugs, are not covered by basic medical insurance in China. Even for those health services that are covered, the out-of-pocket expenses are still quite high. These conventional expenditures have already put a huge strain on us. Receiving TCM care at the hospital is not cheap either. Handling it at home now saves us a lot of money. (P14).

For BCSs who have returned to work or are occupied with household responsibilities, home-based self-care provides greater flexibility in managing their time.

Once my condition was stable, I went back to work. I did not have time to go to the hospital two to three times per week for TCM care. (P12).

Every day, I check my daughter’s homework while giving myself a foot bath with Chinese herbs. (P1).

At home, I can manage my time flexibly—whenever I have a free moment, I can perform self-care. But if I go to the hospital for treatments, I have to set aside a big chunk of time. Even though a treatment like moxibustion only takes 20 min, the waiting and travel time can add up to four or five hours, which means spending a whole morning just for a 20-min treatment. (P3).

*Fieldnotes*: Observations within participants’ homes revealed their adroit utilization of fragmented time for self-care practices. For example, three BCSs engaged in Baduanjin exercises or self-administered moxibustion while waiting for rice to cook or bread to bake. Additionally, two BCSs performed acupressure on their limbs and/or auricular acupoints while responding to interview questions.

#### Home-based treatment setting

Compared to hospital settings, the home environment offered BCSs a more familiar and secure atmosphere. Consequently, home-based self-care facilitated a greater sense of relaxation.

Smelling the disinfectant at the hospital takes me back to the day I was lying on the operating table. It’s a scary memory. Only at home do I feel relaxed. (P2).

I developed insomnia after surgery. At the hospital, during acupuncture and fire cupping, the doctor asked me to close my eyes and try to sleep. However, I was nervous and couldn’t sleep at all because of the noise from other patients in the treatment room. At home, I lie quietly on my bed and listen to the *Five-Elements* TCM music, and sometimes I actually fall asleep. (P10).

*Fieldnotes*: P10 played the *Five*-*Element* TCM music that she had downloaded from the Internet for us. We observed a telling detail: as the music played, her hand gently tapped against the edge of the bed in rhythm with the melody. Occasionally, she closed her eyes, and her face softened into an expression of deep comfort. It seemed she was vividly conveying the sense of security that only comes from being at home through her bodily gestures.

Only a small proportion of BCSs recognized that although home-based self-care offered convenience, the sanitary conditions at home did not adhere to medical standards, and TCM practices conducted without professional medical supervision could pose safety risks.

In the hospital, nurses regularly clean and sanitize the treatment setting and equipment. At home, the cleanliness and sanitation standards definitely don’t match that level. (P22).

The ash from burning moxibustion can easily fall and burn the skin. In the hospital, nurses are there to handle any ash that falls right away. At home, I have to rely on my husband to keep an eye on it, but he is very nervous. If I get burned, we wouldn’t know what to do, so we have to be extra careful. Once, my husband was not paying attention, and the moxibustion ash burned a big hole in the bedsheet. It was indeed dangerous. (P6).

*Fieldnotes*: When recounting this experience, P6 patted her chest with a fearful expression and raised her tone significantly. Her husband interjected, admitting that he actually felt more nervous than his wife during each moxibustion session. To illustrate, he retrieved a moxa stick and showed us the meticulous markings he had inscribed along its length. He explained that these calibrated notches—drawn based on his accumulated observations—indicated critical points where ash buildup typically required immediate removal to prevent burns. During his wife’s self-moxibustion, his gaze would remain fixated on these markers.

#### Immature Internet-Based Sharing TCM Nurse Program

For BCSs preferring a home-based care environment, the Internet-Based Sharing Nurse (IBSN) program presents an alternative option. This service enables patients to book registered nurses for home visits through mobile applications or websites to receive prescribed care services [47]. Although well-established in developed nations such as the United States, Japan, and Australia, IBSN remains in the pilot phase in China [48]. None of the participants had prior experience with IBSN, though some demonstrated interest after learning about the service.

Having a professional nurse come to my home for TCM care is definitely more effective than doing self-care by copying videos. (P20).

However, more participants expressed reservations, indicating their adoption decision would primarily depend on cost considerations, provider qualifications, and post-service support. Furthermore, two elderly BCSs cited usability challenges with the booking applications as a potential barrier.

I choose self-care to save on medical costs. If the IBSN service is too expensive and not covered by insurance, I won’t consider it. (P7).

I only go to tertiary Grade A hospital for fire cupping and moxibustion because their nurses are well-regarded. What about the qualifications of online booked nurse? (P15).

Who should I complain to if there is a medical dispute with the booked nurse? Can the ordering platform help? (P18).

I am not very good with smartphones. If the ordering system is too complicated, I won’t use the service. (P13).

### Capability domain

#### Insufficient knowledge and skills

Limited knowledge and expertise were perceived by participants as the primary obstacle in effective home-based self-care.

My husband and I saw nurses perform fire cupping at the hospital and thought it looked easy, but it turned out to be hard to do ourselves. My husband could not get the cups to stick to my skin at all. (P23).

For moxibustion and massage, you first need to find the right acupoints. Since we have not studied TCM, we cannot locate them accurately. (P5).

*Fieldnotes*: Field observations revealed that while many participants had purchased acupoint charts and auricular models for self-application, they encountered significant difficulties in accurately locating and stimulating the appropriate acupoints. Some participants demonstrated their acupressure techniques, which exhibited notable problems in acupoint selection methodology. Their typical approach involved (1) conducting online searches using symptom-based queries (e.g., “[symptom] AND acupoints”), (2) recording the identified acupoints, and (3) attempting application using their acupoint charts or auricular models. When questioned about verification procedures for acupoint accuracy or resolution of conflicting source information, none could provide adequate methodological solutions.

#### Efficacy uncertainty and safety apprehension

While most participants reported subjective improvement following TCM-based self-care, some questioned whether these perceived benefits reflected actual efficacy or placebo effects influenced by prior expectations.

Every time I get a Gua-sha, I feel completely relaxed, and the next day I wake up feeling much more energized. But I am not sure if it is just a placebo effect. (P10).

Additionally, concerns were raised by some participants regarding potential safety risks associated with the self-care process.

After buying moxa sticks online and using them at home, I had a problem with keeping the right distance. If it’s too far, it does not work; if it’s too close, it burns my skin. (P17).

I initially asked my husband to learn from online videos to do some back Tuina for me, but after hearing news about how improper massage could lead to paralysis, we stopped. (P4).

## Discussion

### Summary of findings

**Fig. 1:**
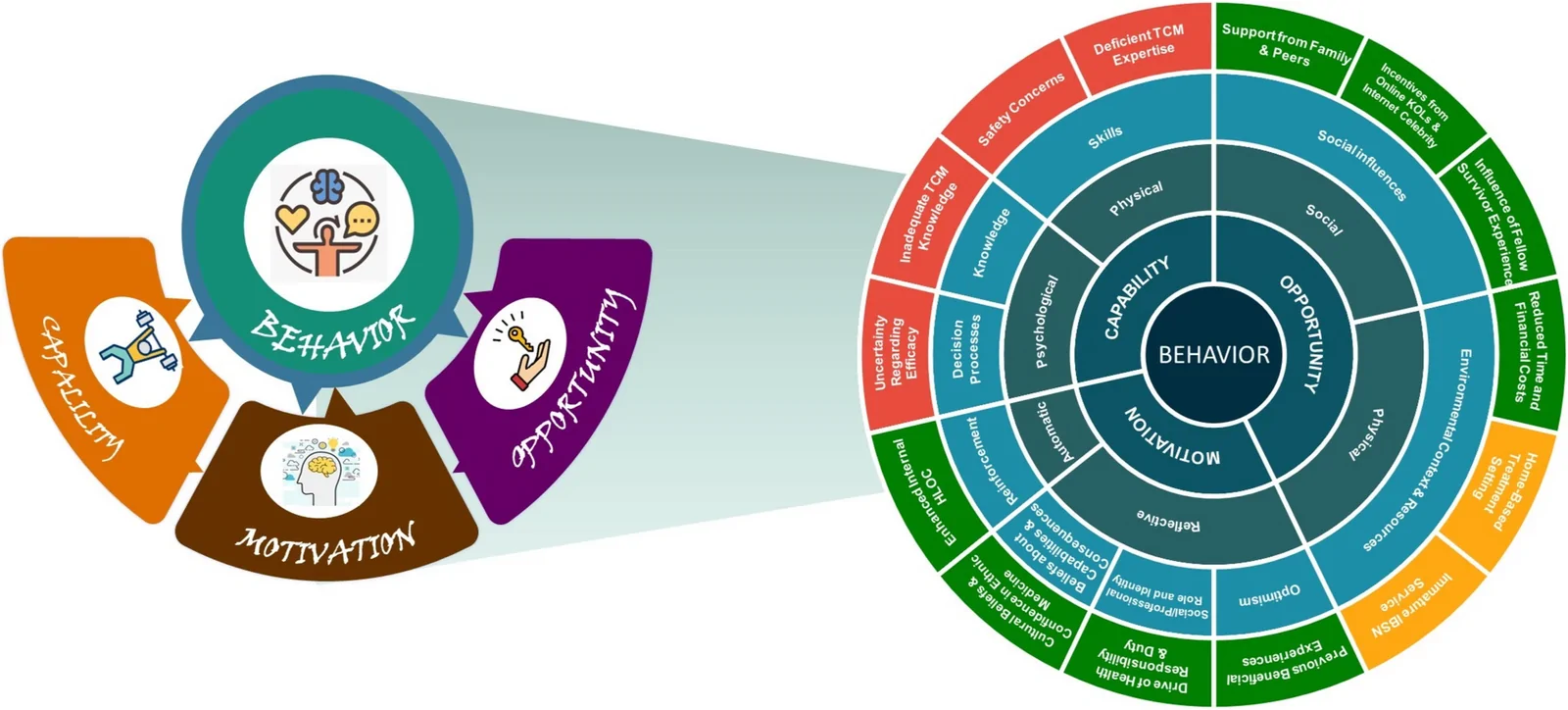
A *behavioral wheel* derived from the COM-B/TDF matrix, mapping factors influencing breast cancer survivors’ engagement in self-directed TCM-based self-care. The outer ring reflects facilitators and barriers identified in the current focused ethnographic study. *GREEN* segments denote facilitators, *RED* segments indicate barriers, and *YELLOW* segments represent factors that function as both. *Abbreviations*: HLOC, health locus of control; KOLs, key opinion leaders; TCM, Traditional Chinese Medicine; TDF, Theoretical Domains Framework. The icons utilized in this figure are adapted from *Vecteezy* and *Flaticon* under the *Creative Commons CC-BY license*

This study investigated TCM-based self-care as a prominent health-cultural phenomenon among BCSs. Key determinants, including barriers and facilitators contributing to this tendency, were systematically identified, and mapped onto a behavioral wheel evolved from the COM-B/TDF matrix (Fig. 1). Their intertwined and co-constructed relationships highlighted the inherently complex and multidimensional nature of this health practice.

### Interpretation of the findings

#### Culturally rooted, belief-driven unique care preferences

Most participants perceived TCM as an intrinsic element of their ethnic cultural identity, attributing their TCM-based self-care practices to profound trust in this ancestral medical tradition. This aligns with established evidence that cultural parameters, particularly values, beliefs, and customs, significantly influence cancer survivors’ health maintenance behaviors [49]. As Cheng et al. reported, Chinese cultural paradigms lead local BCSs typically to associate replenishing *Qi* (*Qi*, pronounced “chee,” denotes the “vital life force” in traditional Chinese philosophy) with their enhanced resistance against cancer recurrence [50]. Among Chinese immigrant communities in Western countries like Canada and Australia, TCM beliefs also shaped their cancer service utilization [51]. Interestingly, such ethnic medicinal beliefs are equally pervasive in regions outside China where CAM is commonly practiced. For instance, approximately 70% of rural Indians consider traditional medicine as primary healthcare [52], while Traditional Korean Medicine has historically been central to Korean prevailing practice and belief systems [53]. These cross-cultural observations highlight the critical need for healthcare providers to incorporate BCSs’ culturally rooted, belief-driven care preferences into clinical decision-making processes.

#### Seeking a holistic cancer recovery pathway

None of the participants abandoned conventional biomedical therapies, demonstrating their trust in mainstream cancer regimens. However, they concurrently pursued self-directed TCM care to mitigate the side effects of orthodox treatments and/or enhance their quality of life during recovery, addressing the “care gap” that Western medicine could not completely bridge. This dual approach underscores TCM’s complementary role in breast cancer survivorship care trajectory. The “care gap” is challenging to quantify, as it extends beyond medical data, survival, or recurrence rates [54]. Instead, it encapsulates the subjective and individual experiences of the survivors, who, due to their reluctance to rely entirely on conventional medicine, sought greater control over their health through TCM-based self-care [54].

We interpret these findings as an awakening or reinforcement of participants’ internal, rather than external, HLOC, reflecting their intention to attribute health outcomes to their own actions rather than being subject solely to fate or luck [55]. The BCSs’ self-help process not only externalizes this awareness but also signifies a heightened sense of responsibility for their own health and well-being [56].

#### Underestimated operational risks and environmental challenges

While BCSs’ sense of health responsibility deserves encouragement, significant safety threats exist in unsupervised home-based TCM self-care.

Participants commonly misjudged moxibustion and fire cupping—their frequently used techniques—as safe for self-administration due to these techniques’ non-invasive nature. However, evidence indicates moxibustion risks include burns, infections, allergies, and hyperpigmentation, with safety contingent upon practitioner proficiency and patient’s condition [57]. Similar hazards accompany fire cupping, necessitating professional administration [58]. For untrained individuals, including BCSs and their families, these practices are far from safe, let alone more invasive treatments like thumb-tack needles and herbal enemas, as noted in interviews. Unguided self-care also raises concerns regarding delayed professional consultation, incorrect self-diagnosis, dangerous drug interactions, inappropriate therapy choices, and masking severe conditions [59].

Safety concerns additionally originate from environmental challenges when utilizing home as a treatment setting. The concentration of air pollutants, such as nitrogen oxides, carbon monoxide, and particulates, during moxibustion can exceed standard levels by tenfold [57]. Residential settings lacking professional ventilation or air purification systems may expose patients to these pollutants. In addition, burns are the most common adverse event associated with moxibustion [57] and the second most common with fire cupping [58]. Such injuries, routinely manageable by nurses in hospitals, may not receive adequate care at home.

#### Forging clinician-BCS therapeutic alliances within collaborative care framework

The IBSN program preserves the spatial and temporal benefits of home-based self-care while addressing safety risks for BCSs. However, it remains in the pilot phase in a few selected Chinese cities [48]. Cost barriers further limit patient uptake, as evidenced by this study and prior chronic disease-related research [48]. Consequently, until more cost-effective IBSN services are developed, TCM-based self-care will likely persist, with the provision of professional guidance and supervision over such practices emerging as the most viable interim solution. This necessitates forging therapeutic alliances between healthcare professionals and BCSs within collaborative care frameworks. Specifically, it is recommended that BCSs engage in home-based TCM self-care under eHealth technologies–based telemonitoring by healthcare professionals to ensure greater safety, while receiving regular professional feedback and symptom interpretations, thereby empowering them to proactively respond to dynamic health status fluctuations [60].

#### Regulating TCM communication and standardizing practices

Many participants who practiced TCM self-care were motivated by social media influencers. Despite lacking formal medical expertise, these online KOLs often demonstrate substantial persuasive power and may, due to the “Halo Effect,” be perceived as more credible than medical professionals [61]. However, reliance on such KOLs risks overlooking the troubling pervasiveness of health-related misinformation on social media [62]. Governments and healthcare organizations bear responsibility to ensure that the public acts upon sound medical advice [61], including rational self-care practices. Critical steps should include coordinated action between health regulatory bodies and social media platforms to strengthen monitoring of online health content and verify the credentials of information providers. Concurrently, with integrative oncology gaining increasing momentum worldwide [56], it is imperative for health authorities to convene experts to develop evidence-based self-care guidelines or consensus recommendations. Such standardized protocols would empower oncologists and nurses to rationally incorporate TCM into BCSs’ routine care while ensuring patients can make adequately informed decisions about safe self-care practices.

Certain limitations should be considered in result interpretation. First, the strict control of interview durations, while necessary for participant welfare, may have restricted the depth of information shared. Second, the study’s modest sample size relative to the extensive variety of TCM modalities (≥ 30 types) presented challenges in achieving comprehensive data saturation during fieldwork observations. Future investigations would benefit from expanded recruitment strategies to better quantify the prevalence and magnitude of identified barriers and facilitators [44]. Third, the exclusively Chinese demographic limits cross-cultural generalizability of findings, particularly given TCM’s growing global utilization. Finally, including perspectives from healthcare providers (oncologists, nurses, TCM practitioners) in subsequent research would provide valuable complementary insights to the current patient-centered findings.

## Conclusion

Chinese BCSs engage in TCM-based self-care for multifaceted reasons, encompassing strong cultural beliefs in ethnic medicine, awakened/intensified internal HLOC, enhanced health responsibility, peers and influencers incentives, prior positive experiences, and time and financial constraints. However, limited TCM knowledge/expertise and infrequent disclosure of TCM use to clinicians contribute to misuse/abuse of such self-care practices and associated medical risks. Within patient-centered framework, healthcare professionals should adequately consider BCSs’ belief-driven unique care preferences within their cultural context. Given the benefits of self-care in breast cancer rehabilitation and the necessity for care continuity, professionally directed TCM-based self-care warrants promotion. This requires healthcare providers to build partnerships with BCSs during the transitional care phase to offer collaborative care. The introduction of telemonitoring technologies in BCSs’ homes creates possibilities for safer and more effective self-care delivery. Beyond strengthening digital health communication governance through social media collaborations, health authorities must prioritize the development of applicable evidence-based clinical practice guidelines or consensus specific to TCM self-care for BCSs.

## Supplementary Information

Below is the link to the electronic supplementary material.

ESM 1 (PDF 595 KB)

## Data Availability

The data that has been used is confidential due to ethical restrictions.

## Abbreviations

BCS(s): Breast cancer survivor(s)
CAM: Complementary and alternative medicine
COM-B: Capability, Opportunity, Motivation-Behavior
COREQ: Consolidated Criteria for Reporting Qualitative Research
HLOC: Health locus of control
IBSN: Internet-Based Sharing Nurse
KOL(s): Key opinion leader(s)
QCA: Qualitative content analysis
SRQR: Standards for Reporting Qualitative Research
TCM: Traditional Chinese Medicine
TDF: Theoretical Domains Framework
