# Supplementary material for: Home-based self-management using Traditional Chinese Medicine techniques: empowerment and risks in breast cancer survivors’ fulfillment of health-deviation self-care requisites
Source: J Cancer Surviv. 2025 May 9;20(Suppl 1):293–304. doi: 10.1007/s11764-025-01808-2 (PMC13676671; doi:10.1007/s11764-025-01808-2)
Supplement: Supplementary file 1 [file 11764_2025_1808_MOESM1_ESM.pdf]

Supplementary Materials

Home-Based Self-Management Using Traditional Chinese Medicine Techniques: Empowerment and Risks in Breast Cancer Survivors' Fulfillment of Health-Deviation Self-Care Requisites

Fei-Yi Zhao[1,2,3,4†], Peijie Xu[5†], Gerard A. Kennedy[3], Li-Ping Yue[1], Wen-Jing Zhang[4], Yan-Mei Wang[4], Yuen-Shan Ho[6*], Qiang-Qiang Fu[7*], Russell Conduit[3]

[1] *Department of Nursing, School of International Medical Technology, Shanghai Sanda University, Shanghai, 201209, People's Republic of China*
[2] *Sydney School of Health Sciences, Faculty of Medicine and Health, The University of Sydney, Camperdown, NSW, 2050, Australia*
[3] *School of Health and Biomedical Sciences, RMIT University, Bundoora, VIC, 3083, Australia*
[4] *Shanghai Municipal Hospital of Traditional Chinese Medicine, Shanghai University of Traditional Chinese Medicine, Shanghai, 200071, People's Republic of China*
[5] *School of Computing Technologies, RMIT University, Melbourne, VIC, 3000, Australia*
[6] *School of Nursing, Faculty of Health and Social Sciences, The Hong Kong Polytechnic University, Hong Kong SAR, People's Republic of China*
[7] *Yangpu Hospital, School of Medicine, Tongji University, Shanghai, 200090, People's Republic of China*

[†] Fei-Yi Zhao (fyzhao@sandau.edu.cn; https://orcid.org/0000-0001-9009-8870) and Peijie Xu contributed equally to this manuscript and are considered co-first authors.

***Correspondence:**

Associate Professor Yuen-Shan Ho, School of Nursing, Faculty of Health and Social Sciences, The Hong Kong Polytechnic University, Hong Kong SAR, People's Republic of China, Tel: (+852) 2766-6410, Fax: (+852) 2364-9663, E-mail: janice.ys.ho@polyu.edu.hk;
and

Associate Professor Qiang-Qiang Fu, Yangpu Hospital, School of Medicine, Tongji University, Shanghai, 200090, People's Republic of China, Tel: (+ 86) 021-6569 0520, Fax: (+ 86) 021-6569 6249; Email: qiangqiang.fu@tongji.edu.cn

APPENDIX 1 COM-B/TDF-informed Interview Outlines

COM-B Construct	COM-B Micro-Construct	TDF Domain	Questions
CAPABILITY	Psychological	Knowledge	• *Can you talk about your understanding of self-care and TCM techniques?*
		Memory, Attention and Decision Processes	• *What TCM techniques do you know about? Which ones do you use at home, and why did you choose those?*
			• *How do you decide on the treatment course and frequency?*
		Behavioural Regulation	• *After being diagnosed with breast cancer, under what circumstances would you consider using home-based TCM techniques for self-care?*
	Physical	Skills, Abilities or Proficiencies	• *Where did you learn how to use these TCM techniques? Did you try them elsewhere before using them at home? If so, how was your experience?*
OPPORTUNITY	Physical	Environmental context and Resources	• *What do you think is the biggest difference between using these TCM techniques in a hospital and at home?* *Prompt—What are the pros and cons of each?*
			• *What factors influence your decision to use or not use TCM-based self-care at home?*
	Social	Social influences	• *How do your family, friends, and healthcare providers feel about your decision to use TCM-based self-care? Do they support or oppose it?*
			• *What do you think about the internet-based sharing TCM nurse services?*
MOTIVATION	Reflective	Beliefs about Capabilities	• *Are you worried that performing TCM techniques incorrectly would weaken the therapeutic effects or even cause safety issues?*
		Beliefs about Consequences	• *After using TCM-based self-care, how have it affected your symptoms and daily life?*
		Social/Professional Role & Identity	• *Do you think that using TCM-based self-care is a beneficial complement to the treatment provided by the hospital?*
		Goals	• *What is your main goal for using TCM-based self-care?*
		Intentions	• *Do you use TCM-based self-care regularly, or only when you feel unwell?*
		Optimism	• *How well do you think using TCM-based self-care helps in achieving your expected health goals?*
	Automatic	Emotion	• *What is the biggest challenge you have faced while using TCM techniques for home-based self-care?*
		Reinforcement	• *Would you give up on using TCM-based self-care if you ran into difficulties or obstacles?*
Summary and conclusion of the interview			• *Is there anything else you would like to share about today's interview topic?*

Abbreviations COM-B, Capability, Opportunity, and Motivation-Behavior model; TDF, Theoretical Domains Framework; TCM, Traditional Chinese Medicine.

APPENDIX 2 Strategies Applied in the Study to Achieve "Four-Dimensions Criteria" Established by *Lincoln* & *Guba* (1985)

Rigour Criteria	Purpose	Strategies Employed
Credibility	Establish confidence that the results are true, credible and believable	• *Ensure all investigators possess the requisite knowledge, skills and research experience for their roles*<br>• *Conduct three preliminary pilot interviews*<br>• *Two investigators independently analyze the data, develop a coding framework, and refine categories*<br>• *Establish a multidisciplinary research team comprising registered nurses, oncologists, TCM practitioners, clinical psychologists, family physicians, and health promotion officers*<br>• *Regularly discuss and debrief research progress with two distinguished academics in the field*
Dependability	Ensure the findings of the qualitative inquiry are repeatable	• *Provide standardized online training to all researchers prior to the study to ensure consistency and quality*<br>• *Record reflective journals after each interview/fieldwork session, including the data collection process*<br>• *Present research findings to participants for comments and validation*<br>• *Determine the final coding structure through consensus in team meetings*<br>• *Meticulously document procedures and decisions at each stage to enhance transparency of the entire research process*
Confirmability	Extend the confidence that the results would be confirmed or corroborated by other researchers	• *Provide transcripts to participants for feedback, including corrections and clarifications*<br>• *Solicit written and oral reflections and feedback from investigators on a regular basis*
Transferability	Enhance the generalizability of the results to other contexts or settings	• *Draw on the "Principles for Specifying Data Saturation" as outlined by Francis et al.*<br>• *Employ iterative sampling method to achieve data saturation*

APPENDIX 3 Sample Characteristics (*n* = 26)

Characteristics	Number of Participants (%)
**Age**	
≤ 45	5 (19.2)
46 - 60	17 (65.4)
> 60	4 (15.4)
**Marital Status**	
Legally Married/Cohabiting	23 (88.5)
Never Married	0 (0)
Divorced	2 (7.7)
Widowed	1 (3.8)
**Educational Level**	
Primary Education or Below	3 (11.5)
Secondary Education	14 (53.8)
Tertiary Education	9 (34.6)
**Employment Status**	
Employed	9 (34.6)
Unemployed/House worker	4 (15.4)
Long-Term Sick Leave	2 (7.7)
Retired	11 (42.3)
**Monthly Per Capita Household Income (Chinese Yuan)**	
≤5,000	4 (15.4)
5,001 - 8,000	14 (53.8)
≥ 8,001	8 (30.8)
**Treatment Type**	
Surgical	26 (100)
Breast-Conserving Surgery	15 (57.7)
Mastectomy	11 (42.3)
Non-Surgical	0 (0)
Other Conventional Treatments (e.g., Radiotherapy, Chemotherapy, Immunotherapy, Targeted Therapy, and Endocrine Therapy, etc.)	24 (92.3)
**Currently Usage Status**	
Still in Use	23 (88.5)
Discontinued	3 (11.5)
**Information Sources of TCM-Based Self-Care**	
Social Media	10 (38.5)
Peer Survivors	9 (34.6)
Families and Friends	5 (19.2)
Mass Media	1 (3.8)
TCM Practitioner	1 (3.8)
Oncologist	0 (0)

Nurse	0 (0)
**TCM Techniques Used**	
Moxibustion	21 (80.8)
Auricular Acupressure	18 (69.2)
Fire Cupping	16 (61.5)
Acupressure	11 (42.3)
Chinese Herbal Foot Bath	9 (34.6)
Medical Diet Therapy of TCM	4 (15.4)
Qigong (e.g., Baduanjin, Tai Chi, etc.)	3 (11.5)
Gua Sha	2 (7.7)
Acupoint Application	2 (7.7)
Five-Elements Music Therapy	1 (3.8)
Retention Enema with TCM	1 (3.8)
**Primary Reasons of Using TCM Techniques**	
Recurrence Prevention	26 (100)
Boost Immunity	22 (84.6)
Increase General Well-Being and Quality of Life	19 (73.1)
Symptoms Managed	19 (73.1)
Fatigue	18 (69.2)
Sleep Disorder	16 (61.5)
Depression/Anxiety	13 (50)
Hot Flashes	12 (46.2)
Chemotherapy-Induced Nausea and Vomiting	12 (46.2)
Night Sweats	10 (38.5)
Dry Mouth	9 (34.6)
Dizziness	9 (34.6)
Loss of Appetite	8 (30.8)
Pain	4 (15.4)
Chemotherapy-induced Constipation	3 (11.5)
Lymphedema	2 (7.7)
Memory Loss	2 (7.7)
Chemotherapy-Induced Peripheral Neuropathy	1 (3.8)
Radiotherapy-Induced Skin Reactions	1 (3.8)

Abbreviations TCM, Traditional Chinese Medicine.

APPENDIX 4 Five-Step Qualitative Content Analysis (QCA) Framework with Exemplar Application

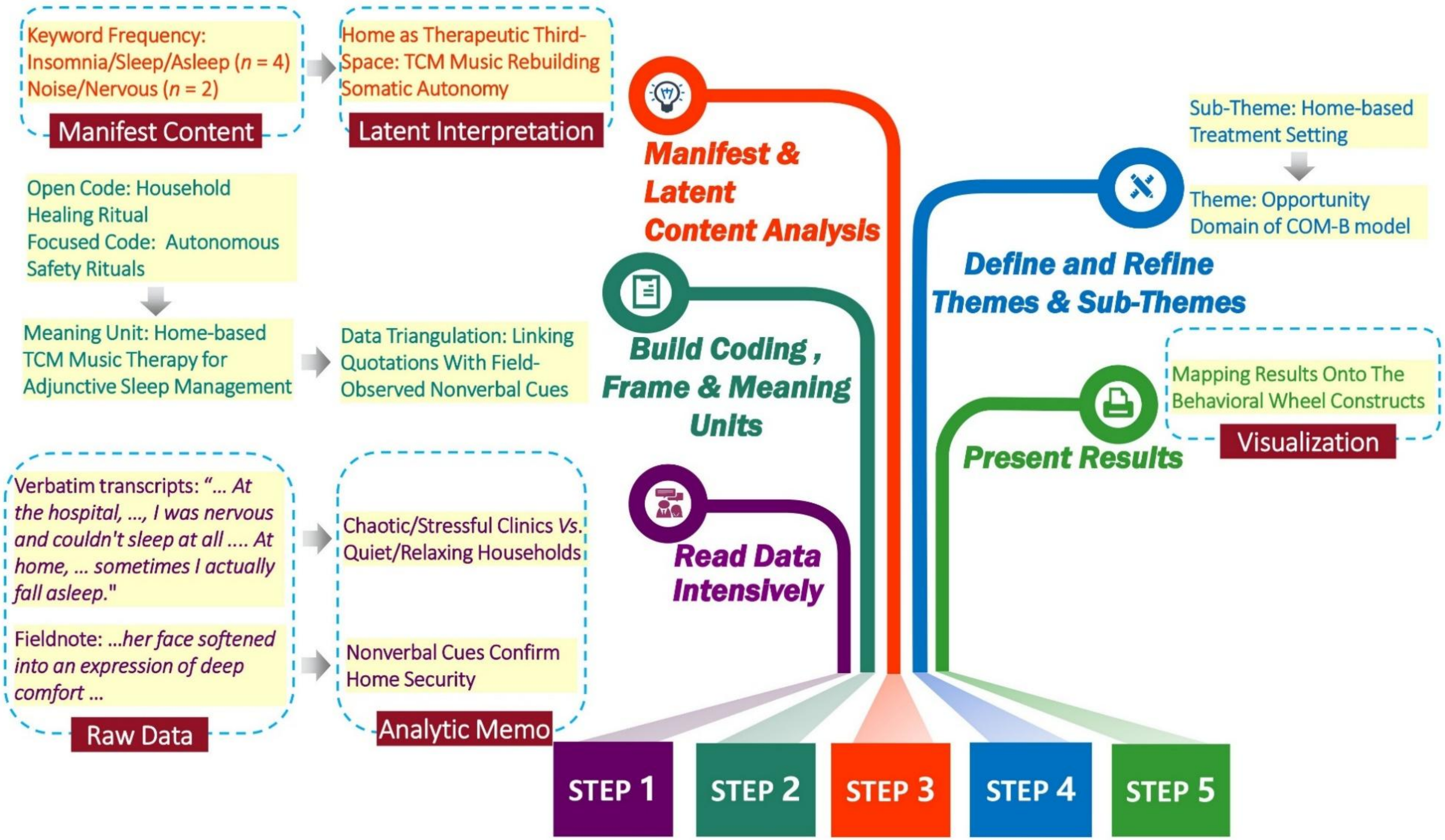

Notes: Main panel outlines the analytical procedure; ***Inset*** demonstrates its implementation through Participant #10's verbal/non-verbal cues.
